# Assessment of Patient Safety and Cultural Competencies among Senior Baccalaureate Nursing Students

**DOI:** 10.3390/ijerph17124225

**Published:** 2020-06-13

**Authors:** Seung Eun Lee, Meen Hye Lee, Anya Bostian Peters, Seok Hyun Gwon

**Affiliations:** 1 Mo-Im Kim Nursing Research Institute and Yonsei University College of Nursing, Seoul 03722, Korea; LEESE@yuhs.ac; 2 School of Nursing, University of North Carolina Wilmington, Wilmington, NC 28401, USA; 3 Susan and Alan Solomont School of Nursing, University of Massachusetts Lowell, Lowell, MA 01854, USA; anya_peters@uml.edu; 4 College of Nursing, University of Wisconsin-Milwaukee, Milwaukee, WI 53211, USA; gwon@uwm.edu

**Keywords:** cultural competency, nursing curriculum, patient safety competency

## Abstract

This descriptive, correlational, cross-sectional study examined nursing students’ educational experiences on self-reported perceptions of patient safety and cultural competence in terms of curriculum content and learning venues. We performed descriptive analyses and a one-way analysis of variance with a sample of senior-year nursing students (N = 249) attending three state universities in the United States. We used the Nurse of the Future Nursing Core Competency Model, the Patient Safety Competency Self-Evaluation Tool for Nursing Students, and The Cultural Competence Assessment Instrument. Overall, participants reported that patient safety and cultural competencies were addressed in their curricula primarily through classroom activities as opposed to laboratory/simulation or clinical settings. Among the required patient safety knowledge topics, elements of highly reliable organizations were covered the least. For patient safety competency, participants reported higher scores for attitude and lower scores for skill and knowledge. For cultural competency, participants scored much higher for cultural awareness and sensitivity than behavior. There was no statistically significant difference between scores for patient safety and cultural competencies by nursing school. The results support the need for curriculum development to include all important aspects of patient safety and cultural competencies in various teaching/learning venues.

## 1. Introduction

Quality problems are pervasive in healthcare and affect every patient, so enhancing the quality of care is essential. The Institute of Medicine challenges healthcare professionals to provide high-quality care [1] while the International Council of Nursing (ICN) declares that patient safety is fundamental to quality health and nursing care [2]. Therefore, healthcare professionals ensure safe, effective, patient-centered, timely, efficient, and equitable care to provide high-quality care [1]. Improving the quality of healthcare enhances overall patient health and outcomes but depends upon the avoidance of care-related injuries as well as responsiveness and respect for patient needs and values. This means the healthcare workforce must be well prepared to ensure patient safety and provide culturally sensitive care [3,4]. 

Providing safe care is an essential responsibility of hospital nurses, as patients in that setting are at risk of adverse events (e.g., medication administration errors, falls, pressure ulcers, and pneumonia), many of which are related to nursing duties [5,6]. Additionally, the United States’ population is undergoing a significant demographic shift, as minority populations are projected to comprise half the general population by 2050 [7]. The increasing ethnic and cultural diversity calls for greater cultural competence. This, and patient safety awareness, is particularly important for nurses who provide direct care to patients on the front line of contemporary healthcare environments [8]. Patient safety competency is defined as the ability to minimize risk of harm to patients through individual performance and implementation of effective systems [9], and cultural competency refers to the ability to provide high-quality, culturally sensitive care [10]. 

The ICN asserts that patients have the right to culturally and clinically appropriate care and advocates that nurses should: exhibit cultural competence by being aware of their own culture without letting it unduly influence those from other backgrounds; demonstrate understanding of different cultures; accept that differences between cultural beliefs may exist between the patient and the provider; accept and respect cultural differences; modify care to be compatible with the patient’s culture and expectations; and provide culturally appropriate care that will ensure best patient outcomes [11]. Preparing nurses to provide safe and culturally competent nursing care requires significant education. That is, nurses must be made fully aware of an array of patient safety issues as well as preventive measures, and they must clearly understand the importance of being responsive to each patient’s beliefs and values, religion, primary language, and other cultural factors in order to make culturally sensitive decisions [10]. Inculcating nurses with knowledge that transcends geographic boundaries is essential to providing culturally competent care and decreasing health disparities [12,13]. The means of providing safe and culturally competent quality care should be central components of nursing education [14]. Nurses with these competencies are better able to integrate knowledge and skills with appropriate attitudes to provide safe and quality care to diverse patient populations.

As patient advocates, nurses are in a pivotal position to effectively provide safe, culturally competent care. Cultural competence increases access to quality care for all patient populations and would replace generic care delivery models with a system that is more responsive to diverse populations. Cultural competence can also establish rapport with patients. However, culturally competent care cannot be delivered unless nurses and other healthcare professionals have a well-defined understanding of diverse cultural backgrounds [15]. Although increased interest and effort have been devoted to developing curricula adequately addressing patient safety and cultural competencies [4,10], there is a lack of consensus on how to integrate these competencies into nursing programs [4]. The National League for Nursing introduced the initiative, “The Power of Diversity: Embracing Differences in Heritage and Thought”, and appealed for action in “Creating Inclusive Academic Environments”, which would foster a robust and diverse nursing workforce. The recommendation for nursing curricula would facilitate students providing culturally appropriate health care with attentiveness to health disparities in valuing diversity and inclusivity of increased populations [16]. 

A review of 17 studies on undergraduate nursing education found only a limited number described specific patient safety and cultural competency learning requirements [17]. This suggests that nursing students may be inadequately prepared to provide quality care that maximizes patient safety [18]. Furthermore, little is known about nursing students’ educational experiences with respect to patient safety and cultural sensitivity and their perceptions of their competencies in these areas. Better understanding of experiences and perceptions will enable educators to better design curricula that instill these competencies in future nurses. Therefore, the objectives of this study were to assess nursing students’ perceptions of patient safety and cultural competency-related educational experiences in terms of curriculum content and learning venues and to examine their self-reported patient safety and cultural competencies.

## 2. Materials and Methods 

### 2.1. Study Design and Sample

This quantitative study adopted a descriptive, correlational, and cross-sectional design. This study took place in three state universities in the Northeast, Midwest, and Southeast United States. Each institution provides a baccalaureate nursing program and all senior students enrolled in the program at the time of data collection were eligible to participate in this study, using a convenience sampling method. The inclusion criteria were 1) full-time, senior-year nursing students, 2) 18 years of age or older, and 3) agreed to participate in this study. At each school, a primary faculty contact provided study information to eligible students, explaining that participation was voluntary and they had the right to withdraw from the study at any time without penalty. A hard-copy self-report survey was administered and submittal of a completed hard-copy survey expressed consent. A total of 249 senior-year nursing students completed the survey from July to October 2018, representing a response rate of 94%. Ethics approval was obtained from the primary investigator’s university (School A: #18-012-LEE-XPD), and collaborating agreements (School B: #19.022 and School C: #00001588) were obtained from the other two universities.

### 2.2. Measures

A survey was utilized to gather participants’ demographic information: age, gender, race/ethnicity, birth country, language spoken at home, and educational institution. The same survey included items to measure participants’ patient care experiences outside their nursing education program as a patient care technician or certified nursing assistant and the duration of such experience. 

The Nurse of the Future Nursing Core Competency Model guided the assessment of students’ cultural and patient safety-related educational experience in terms of curriculum content and learning venues [19]. The model, which guides nursing curricula and practice, includes 10 nursing core competencies and the knowledge, attitudes, and skills related to each. Nursing knowledge is the core of the model [19] and only the knowledge section of patient safety and patient-centered care competencies was used to examine the coverage of content related to the two competencies. Examples of patient safety competency-related content include *processes used in understanding causes of error and in allocation of responsibility and accountability* and *benefits and limitations of commonly used safety technology.* An example of patient-centered care competency-related content is *how human behavior is affected by socioeconomics, culture, race, spiritual beliefs, gender identity, sexual orientation, lifestyle, and age*. The contents were reviewed by five nurse educators in clinical and academic settings for their appropriateness to the target population and their clarity of wording. Study participants were asked whether and how each of the competency topics was covered. Participants chose from these categorical response options: classroom, lab/simulations, course assignments/readings, clinical experiences, and not covered. 

Patient safety competency was measured using the Patient Safety Competency Self-Evaluation Tool for Nursing Students (PSCSE) [18]. This instrument has 45 items in three subscales: knowledge, skills, and attitudes. Items are rated on a 5-point Likert scale ranging from (1) *not knowledgeable* to (5) *very knowledgeable* for the knowledge subscale, (1) *very uncomfortable* to (5) *very comfortable* for the skills subscale, and (1) *strongly disagree* to (5) *strongly agree* for the attitudes subscale. A mean score for each subscale was calculated as recommended by the instrument developers; a higher mean score for a subscale indicated a greater level of patient safety competency within the dimension. The internal consistency and construct validity of the PSCSE have been established [18]. For the study sample, the Cronbach’s alpha scores for the total PSCSE and the knowledge, skills, and attitudes dimensions were 0.87, 0.71, 0.85, and 0.73, respectively. 

Cultural competency was measured using the Cultural Competence Assessment Instrument (CCA) [20]. The CCA has 25 items in two subscales: cultural awareness and sensitivity and cultural competence behaviors. The instrument uses a 7-point response scale ranging from (1) *strongly disagree* to (7) *strongly agree* for the cultural awareness and sensitivity subscale and (1) *never* to (7) *always* for the cultural competence behaviors subscale. A higher score on the CCA indicates a higher level of cultural competence. The instrument’s reliability and validity have been established [20]. For the study sample, Cronbach’s alpha scores for the total CCA, cultural awareness and sensitivity subscale, and cultural competence behaviors subscale were 0.86, 0.70, and 0.90, respectively. 

### 2.3. Data Analysis

Descriptive statistics (mean, standard deviation, and percentage) were used to describe the sample’s demographic characteristics and key study variables. After checking for normality of distribution of each outcome variable by examining histograms and Q-Q plots and homogeneity of variances by conducting Levene’s test, a one-way analysis of variance (ANOVA) was used to test for differences in nursing students’ perceptions of patient safety and cultural competency levels by university and by student characteristics. All statistical analyses were completed using STATA Version 15 (StataCorp, College Station, TX, USA). In all analyses, the statistical significance level was *p* < 0.05.

## 3. Results

### 3.1. Sample Characteristics

Table 1 presents the characteristics of the study sample. A majority of participants were female (87.15%) with an average age of 22.66 years. Most participants were White (87.60%), were born in the United States (92.37%), and spoke English at home (86.75%). Approximately 64% of the participants had patient care experiences as a patient care technician or certified nursing assistant outside their nursing education program, with a mean experience duration of 1.86 years.

### 3.2. Patient Safety Competency-Related Educational Experience

As shown in Table 2, variations were found in participants’ educational experience with respect to the 10 patient safety competency-related knowledge topics. Overall, participants reported that the topics were primarily covered in the classroom and secondly during clinical experiences. Of the 10 topics, *human factors and basic safety design principles that affect safety* was most frequently covered, followed by *factors that create culture of safety* and *how patients, families, individual clinicians, health care teams, and systems can contribute to promoting safety and reducing error.* Notably, more than 20% of participants reported that three of the 10 topics were not covered in their nursing programs. Specifically, about 50% reported that *elements for sustaining a high reliable organization* were not covered, and about 20% each reported that *effective strategies to enhance memory and recall and minimize interruptions* and *process used in understanding causes of error and in the allocation of responsibility and accountability* were not addressed. 

### 3.3. Patient Safety Competency

The mean PSCSE score for participants’ overall patient safety competency was 3.94 of 5 points (Table 3). The attitudes subscale showed the highest mean score at 4.18 (*SD* = 0.28), followed by the skills (*M* = 3.88, *SD* = 0.49) and knowledge (*M* = 3.77, *SD* = 0.55) subscales. Based on ANOVA results, these mean scores did not significantly differ by nursing school. No statistically significant differences were found in mean scores for participant-reported patient safety competency based on prior patient care experiences as a patient care technician or certified nursing assistant. 

### 3.4. Cultural Competency-Related Educational Experience

Overall, about 98% of participants reported that the three cultural competency-related knowledge topics were covered in their nursing programs (Table 2). All three topics were most frequently covered in the classroom, followed by during clinical experiences and in course assignments/readings. The topics were least addressed in lab/simulation sessions, with rates ranging from approximately 27% to 40%.

### 3.5. Cultural Competency

As shown in Table 3, the average CCA score for participants’ overall cultural competency was 5.33 of 7 points (*SD* = 0.61). The mean score for cultural awareness and sensitivity was 6.02 (*SD* = 0.47), and the mean score for cultural competence behaviors was 4.64 (*SD* = 1.05). ANOVA results revealed that the mean scores for overall cultural competency, cultural awareness and sensitivity, and cultural competence behaviors did not significantly differ between nursing schools. Also, no statistically significant differences were found in mean scores for participant-reported cultural competency based on prior patient care experience as a patient care technician or certified nursing assistant. 

## 4. Discussion

This study examined nursing students’ patient safety and cultural competence-related educational experiences in terms of curriculum content and learning venues and assessed their self-reported patient safety and cultural competencies. Overall, nursing students reported that these competencies were addressed in their curriculums. Patient safety and cultural competence-related knowledge topics were mostly covered in the classroom setting and were less covered in lab/simulation sessions. Similar to the findings of earlier research [4,21], several patient safety and cultural competency knowledge topics were addressed in more than one learning venue. 

With regard to patient safety competency, study participants had higher scores for attitudes and lower scores for skills and knowledge. These findings are also consistent with earlier research [3]. These results indicate that students’ perceptions of the importance of patient safety were higher than their views of their clinical preparedness or actual knowledge [3]. Study results also revealed that patient safety competency-related education was delivered less frequently in lab/simulation and clinical settings than in the classroom. Given the magnitude of patient safety issues in healthcare settings, patient safety cannot be emphasized enough in nursing education. In fact, patient safety needs to be covered within integrated nursing curricula and in multiple learning modes, including simulations, assignments, readings, and clinical experiences. Several researchers have recommended teaching patient safety through simulations for undergraduate nursing students [4,22,23], and specific patient safety scenarios (e.g., involving medical error) can be incorporated into assignments, readings, and other learning activities. Through these activities, students can be challenged to identify patient safety problems, apply their critical thinking skills in finding solutions, and consider future preventive measures. Nursing educators can also consider use of visual learning methods for patient safety topics, as younger generations of nursing students are accustomed to and enjoy acquiring information through visual media. For example, educators could consider creating YouTube video clips showing role-playing to address patient safety topics. Furthermore, clinical courses and practicums need to have specific course objectives for patient safety-related learning.

Notably, approximately half the participants reported that the knowledge topic *elements for sustaining a high reliable organization* was not covered (Table 2). This is a serious deficiency, as understanding the characteristics of a high reliability organization (HRO) is necessary to provide safe quality care to patients. HROs are organizations that operate under complex and hazardous circumstances for long periods without serious accidents [24]. Although healthcare is a complex, unpredictable, and interdependent system, reliability can be achieved by embracing HRO characteristics such as simplifying and standardizing operational tasks and giving decision-making authority to knowledgeable experts, including front-line staff [25]. Educating nursing students about HRO elements is necessary, as nurses should be able to apply these elements when delivering care to their patients [19]. For example, HROs encourage front-line staff to recognize and report small problems (e.g., abnormal vital signs) before they become big problems (e.g., patient adverse events). Therefore, nursing students should be taught how to identify unsafe conditions and risks of potential error, what to report, and how to communicate with leaders about risks, and they should understand the potential organizational consequences of their reports [26]. Thus, nursing educators should review existing curriculums and revise them as necessary to incorporate HRO elements.

Study findings indicated that pedagogical changes are warranted to improve the cultural behaviors of nursing students. Although the participants scored relatively high for perceived cultural awareness and sensitivity, their mean score for cultural competence behaviors was much lower, suggesting that nursing programs are not adequately addressing these behaviors in terms of curriculum content and learning venues. Considering the complexity of cultural competence, a comprehensive and cumulative educational process is needed to integrate delivery of didactic content with opportunities to apply culturally competent behaviors in diverse learning venues [10,27]. Simulation is a promising teaching strategy for fostering cultural competency and cultural competence behaviors in undergraduate nursing students, as simulation technology can facilitate realistic encounters with diverse patient cultures [28]. Indeed, several recent studies have reported positive outcomes for use of simulation to develop cultural competency in undergraduate and graduate nursing students in clinical and community settings [29,30,31,32]. These studies implemented various simulation strategies, including use of an exemplar video and simulated interview with an African-American standardized patient [29], poverty simulation in a community setting [30], use of culturally diverse simulation scenarios for alcohol and drug use [31], and development of a virtual simulation case for patients with diabetes in a community setting [32]. Given the positive results produced by these strategies, nursing educators should consider integrating cultural competency learning opportunities into their use of simulation in order to immerse students in diverse cultural contexts.

Another strategy for fostering cultural competency in undergraduate nursing students is development of clinical and community partnerships. Frequently, affiliations between schools of nursing and clinical facilities exist in largely homogeneous communities, and thus students’ clinical exposure to patient diversity may be limited depending on their school’s location, the hospitals in the community, and the presence of a diverse population. However, intentional design of clinical and community partnerships can allow nursing students to access and provide care for patients with diverse cultural backgrounds. Such exposure serves to solidify students’ knowledge, skills, and attitudes regarding patients of different cultures, dispelling the notion that culture is just “out there” and is not necessarily relevant to practice [33]. In addition, recruitment and retention of faculty with diverse cultural backgrounds support development of culturally competent nursing students. On the same note, nursing schools should strive to recruit and retain culturally diverse preceptors and clinical faculty.

Greater consensus is needed regarding the content and venues that nursing programs should use to instill the knowledge and skills students require to provide safe, culturally competent care. Nursing educators should revisit their curricula, re-evaluate the required patient safety areas least covered, and strengthen the associated content. Furthermore, nursing educators should make it their personal mission to increase students’ cultural awareness, encounters, and competencies in order to optimize their learning experience and prepare them to practice in an increasingly diverse society [34]. To build both patient safety and cultural competencies in their students, nursing programs should employ all the instructional methods available to develop the knowledge, skills, and attitudes students need to provide safe and culturally sensitive care to patients. 

### Limitations

This study had three notable limitations. First, although participants were recruited at three geographically disparate universities, the study’s use of convenience sampling limited the generalizability of the findings. There is also potential selection bias due to non-randomly selected samples. The study’s cross-sectional, correlational design limited the ability to draw causal inferences. Finally, all data were collected using self-report questionnaires, which are inherently subjective.

## 5. Conclusions

To provide safe and culturally sensitive care to patients, nurses must be equipped with patient safety and cultural competencies. Study participants reported that patient safety and cultural competency topics were less frequently covered in lab/simulation and clinical settings than in classrooms and that some patient safety topics were omitted altogether in their programs. Thus, study findings indicate that patient safety and cultural competency-related content was not sufficiently addressed in some learning venues. Nursing faculty need to ensure that their curricula cover all required elements of patient safety and cultural competencies and that these elements are adequately addressed in all available learning venues. 

## Figures and Tables

**Table 1:** Demographic Characteristics of Participants (*N* = 249).

Characteristic	Mean (*SD*)	*n* (%)
Age (years)	22.85 (4.54)	
Gender		
Male		32 (12.85)
Female		217 (87.15)
Race/ethnicity ^a^		
White/Caucasian/European American		212 (87.60)
Black/African American		10 (4.13)
American Indian/Alaska Native		2 (0.83)
Asian		11 (4.55)
Arab American/Middle Eastern		3 (1.24)
Other		4 (1.65)
Language used at home		
English		216 (86.75)
Other		33 (13.25)
Born in the United States		
Yes		230 (92.37)
No		19 (7.63)
Previous patient care experience		
Yes		159 (63.86)
No		90 (36.14)
Previous patient care experience (years)	1.89 (1.93)	

Note: ^a^ There were seven missing responses for race/ethnicity; SD = Standard deviation.

**Table 2:** Patient Safety and Cultural Competency Content Covered and Teaching Venues (*N* = 249)

	Leaning Venue ^a^	Covered *n* (%)	Classroom *n* (%)	Lab/Simulations *n* (%)	Assignments/Readings *n* (%)	Clinical Experiences *n* (%)	Not Covered *n* (%)
Content							
**Patient safety competency**							
Human factors and basic safety design principles that affect safety		244 (97.99)	224 (91.06)	155 (63.01)	145 (58.94)	201 (81.71)	5 (2.01)
Benefits and limitations of commonly used safety technology		237 (95.18)	172 (69.92)	135 (54.88)	96 (39.02)	195 (79.27)	12 (4.82)
Effective strategies to enhance memory and recall and minimize interruptions ^b^		199 (79.92)	160 (65.04)	78 (31.71)	85 (34.55)	118 (47.97)	50 (20.08)
General categories of errors and hazards in care		237 (95.18)	210 (85.37)	121 (49.19)	123 (50.00)	154 (62.60)	12 (4.82)
Factors that create a culture of safety		242 (97.19)	222 (90.24)	151 (61.38)	161 (65.45)	194 (78.86)	7 (2.81)
Optimal processes for communicating with patients/families experiencing adverse events		225 (90.36)	168 (68.29)	122 (49.59)	110 (44.72)	183 (74.39)	24 (9.64)
How patients, families, individual clinicians, healthcare teams, and systems can contribute to promoting safety and reducing errors		242 (97.19)	211 (85.77)	129 (52.44)	150 (60.98)	192 (78.05)	7 (2.81)
Processes used in understanding causes of error and in allocation of responsibility and accountability ^b^		199 (79.92)	183 (74.39)	89 (36.18)	108 (43.90)	132 (53.66)	50 (20.08)
Potential and actual impact of established patient safety resources, initiatives, and regulations		232 (93.17)	188 (76.42)	101 (41.06)	120 (48.78)	167 (67.89)	17 (6.83)
Elements for sustaining a high reliable organization ^b^		125 (50.20)	118 (47.97)	41 (16.67)	70 (28.46)	83 (33.74)	124 (49.8)
**Cultural competency**							
How cultural diversity, ethnic, spiritual, and socioeconomic backgrounds function as sources of patient, family, and community values		246 (98.80)	232 (93.17)	97 (39.43)	168 (68.29)	186 (74.70)	3 (1.20)
How human behavior is affected by socioeconomics, culture, race, spiritual beliefs, gender identity, sexual orientation, lifestyle, and age		245 (98.39)	221 (88.76)	82 (33.33)	158 (64.23)	175 (71.14)	4 (1.61)
The effects of health and social policies on persons from diverse backgrounds and cultures		243 (97.59)	216 (86.75)	66 (26.83)	153 (62.20)	146 (59.35)	6 (2.41)

Note: ^a^ Multiple answers to a multiple choice question; ^b^ More than 20% of student participants reported that the content was not covered in their nursing program.

**Table 3:** Students’ Patient Safety and Cultural Competencies by Nursing School.

Competency Variable	School	*n*	Mean (SD)	F	*p* Value
**Overall patient safety competence**	**All**	**244**	**3.94 (0.34)**	**–**	**–**
	School A	75	4.00 (0.32)	2.80	0.06
	School B	81	3.86 (0.36)	–	–
	School C	88	3.95 (0.33)	–	–
**Patient safety knowledge**	**All**	**245**	**3.77 (0.55)**	**–**	**–**
	School A	76	3.87 (0.55)	2.60	0.08
	School B	81	3.67 (0.58)	–	–
	School C	88	3.77 (0.52)	–	–
**Patient safety skills**	**All**	**245**	**3.88 (0.49)**	**–**	**–**
	School A	75	3.92 (0.45)	0.92	0.40
	School B	82	3.82 (0.53)	–	–
	School C	88	3.89 (0.48)	–	–
**Patient safety attitudes**	**All**	**247**	**4.18 (0.28)**	**–**	**–**
	School A	76	4.21 (0.23)	2.78	0.06
	School B	83	4.12 (0.29)	–	–
	School C	88	4.12 (0.30)	–	–
**Overall cultural competence**	**All**	**245**	**5.33 (0.61)**	**–**	**–**
	School A	76	5.31 (0.62)	0.60	0.55
	School B	82	5.29 (0.62)	–	–
	School C	87	5.39 (0.59)	–	–
**Cultural awareness and sensitivity**	**All**	**246**	**6.02 (0.47)**	**–**	**–**
	School A	76	6.05 (0.45)	2.85	0.06
	School B	83	5.92 (0.50)	–	–
	School C	87	6.08 (0.44)	–	–
**Cultural competence behaviors**	**All**	**246**	**4.64 (1.05)**	**–**	**–**
	A	76	4.57 (1.09)	0.33	0.72
	B	82	4.67 (1.07)	–	–
	C	88	4.70 (1.00)	–	–

Note: Sample sizes vary for each outcome variable due to missing responses; SD = Standard deviation.
